# A population-based study on incidence trends of myeloma in the United States over 2000–2020

**DOI:** 10.1038/s41598-023-47906-y

**Published:** 2023-11-24

**Authors:** Seyed Ehsan Mousavi, Mehran Ilaghi, Armin Aslani, Zahra Yekta, Seyed Aria Nejadghaderi

**Affiliations:** 1 Neurosciences Research Center, Aging Research Institute, Tabriz University of Medical Sciences, Tabriz, Iran; 2 Department of Community Medicine, Social Determinants of Health Research Center, Faculty of Medicine, Tabriz University of Medical Sciences, Tabriz, Iran; 3 Institute of Neuropharmacology, Kerman Neuroscience Research Center, Kerman University of Medical Sciences, Kerman, Iran; 4 Calaveras Department of Public Health, Calaveras County, CA USA; 5 School of Medicine, Shahid Beheshti University of Medical Sciences, Tehran, Iran; 6 Systematic Review and Meta‑analysis Expert Group (SRMEG), Universal Scientific Education and Research Network (USERN), Tehran, Iran

**Keywords:** Cancer epidemiology, Myeloma

## Abstract

Myeloma is one of the most common types of haematological malignancies. We aimed to investigate the incidence rates of myeloma by sex, race, age, and histological subgroups in the United States (US) over 2000–2020. Data were retrieved from the the Surveillance, Epidemiology, and End Results (SEER) 22 database. The International Classification of Diseases for Oncology version 3 morphological codes 9731, 9732, and 9734 were assigned for solitary plasmacytoma of bone, plasma cell myeloma, and extraosseous plasmacytoma, respectively. Average annual percent change (AAPC) and the pairwise comparison with the parallelism and coincidence were reported. All estimates were reported as counts and age-adjusted incidence rates per 100,000 individuals. Over 2000–2019, most of myeloma cases were among those aged at least 55 years (85.51%), men (54.82%), and non-Hispanic Whites (66.67%). Among different subtypes, plasma cell myeloma with 193,530 cases had the highest frequency over the same period. Also, there was a significant decrease in the age-standardized incidence rate of myeloma across all races/ethnicities in both sexes within all age groups (AAPC: − 8.02; 95% confidence interval (CI): − 10.43 to − 5.61) and those aged < 55 (AAPC: − 8.64; 95% CI − 11.02 to − 6.25) from 2019 to November 2020. The overall trends of myeloma incidence rates were not parallel, nor identical. There was an increase in myeloma incidence in both sexes, with a highly increasing rate, particularly among younger Hispanic and non-Hispanic Black women over 2000–2019. However, a remarkable decline was observed in the incidence rates following the COVID-19 pandemic in 2020.

## Introduction

Myeloma is a cancer affecting mature plasma cells which is typically preceded by a pre-cancerous condition called monoclonal gammopathy of undetermined significance^1^. It is the second most prevalent type of blood cancer, accounting for approximately 1% of all cancer cases, 2% of cancer-related deaths, and 12–15% of oncological and hematological diseases^2–6^. This condition can progress through stages of asymptomatic/smoldering myeloma before reaching the stage of overt myeloma^1^. Plasma cell myeloma is the most advanced and aggressive form of myeloma^1,2^. Two distinct types of localized plasma cell myelomas have been identified as solitary plasmacytoma of bone and extraosseous plasmacytoma, which constitutes approximately 4–5% and 1–3% of all plasma cell myelomas, respectively^7^.

The occurrence of myeloma has shown a rise in the United Kingdom, the United States (US), and Western Europe that can be due to improved access to healthcare services and enhanced diagnostic methods^8^. It is influenced by sex, race/ethnicity, and age, with a higher frequency observed among older individuals^9^. Men are more likely to develop the disease than women^1^. Furthermore, Hispanics are diagnosed with myeloma at a younger age and have survival rates comparable to those of non-Hispanic White (NHW) and non-Hispanic Black (NHB)^10^. A recent study showed ongoing advancements in the survival of patients with myeloma and the difficulty of enhancing long-term outcomes, particularly for older individuals and minority populations^4^.

The COVID-19 pandemic had substantial effects on the diagnostic process and treatment administration for myeloma. In this regard, a noticeable decline in the incidence of newly diagnosed myeloma cases in 2020 was demonstrated compared to previous years^11,12^. Also, it is estimated that between March 2020 and February 2021, there were 430,000 fewer patients referred for suspected cancers compared to the previous year^13^.

A previous study has reported the survival and mortality rate of plasma cell myeloma over the period of 1973–2014^14^; however a more precise investigation of updated incidence trends of myeloma seems necessary. Therefore, we aimed to report the incidence trends of myeloma by age, sex, race/ethnicity and histological subgroups over 2000–2020 in the US using the The Surveillance, Epidemiology, and End Results (SEER) database. We also examined how the COVID-19 pandemic affected the overall incidence trends of myeloma.

## Methods

### Data sources

The SEER Programme of the National Cancer Institute is a comprehensive population-based source of cancer information in the US. SEER 22 covers about 48% of the US population and offers patient survival rates and cancer stage information at the time of diagnosis^15^. The SEER programme gathers information on patients' demographics, initial tumour site, tumour morphology, stage at diagnosis, first course of treatment, and vital status follow-up^15^. We used the SEER 22 database, which was released in April 2023 based on data submitted in November 2022, to estimate the incidence rates and annual percent changes (APCs) of myeloma cancer from 2000 to 2020^16,17^. The SEER 22 database was accessed in accordance with the SEER Research Data Agreement for 1975–2020 Data (November 2022 Submission)^18^ and cancer statistics reported according to SEER 22 guideline^19^.

### Definitions

Cancer cases are reported in terms of frequencies and percentages, with the incidence rate given in terms of cases per 100,000 people. The APCs of myeloma cancer for a specific time period indicate rate variation at a consistent fraction of the previous year's rate. The average annual percent changes (AAPCs) described the average of various APCs across the time. Hispanic, NHW, and NHB were classified. Due to the small sample size, the race and ethnicity groups of American Indian/Alaska Native, Native Hawaiian, and Asian/Pacific Islander cases were only used to calculate the parameters of all races and ethnicities. The patients with myeloma cancer were identified based on the International Classification of Diseases for Oncology version 3 (ICD-O-3). "According to the World Health Organization classification, "plasma cell neoplasm" serves as a broad category encompassing various conditions like monoclonal gammopathy of unknown significance, plasma cell myeloma, solitary plasmacytoma of bone, immunoglobulin deposition disease, extraosseous plasmacytoma, and osteosclerotic myeloma, while only plasma cell myeloma, solitary plasmacytoma of bone, and extraosseous plasmacytoma had reportable data"^20^. In this report, we used "myeloma" as the general term for different types of myeloma. The morphologies of myeloma cancer were classified as plasma cell myeloma (ICD-O-3 histologic code 9732), solitary plasmacytoma of bone (ICD-O-3 histologic code 9731), and extraosseous plasmacytoma (ICD-O-3 histologic code 9734), and they were also reported seperately.

### Statistical analysis

To estimate the delay age-standardized incidence rate (ASIR) of myeloma, the SEER 22 Research Limited-Field Data with Delay-Adjustment database for the period from 2000 to 2020^16^ was collected from SEER*Stat, version 8.4.1.2^21^. The purpose of modelling reporting delay is to update the current case count to account for expected future data revisions (including additions and deletions)^22^. These adjusted counts, as well as the associated delay model, are useful in detecting current cancer trends more precisely^22^. The case selection was limited to malignant myeloma cancer diagnosed with a known age. The delay model was then run using delay adjustment factors such as cancer site, registry, age group, race and ethnicity, and the year of diagnosis^23,24^. In addition, to estimate the ASIRs of myeloma subtypes, the SEER 22 Research Limited-Field Data database for the period from 2000 to 2020^17^ was retrieved from SEER*Stat, version 8.4.1.2^21^. The SEER*Stat version 8.4.1.2^21^ was used to estimate the ASIRs based on the 2000 US standard population and the accompanying 95% confidence intervals (CIs) with the Tiwari method^25^.

The Joinpoint Regression Program, version 5.0.2^26^, was employed to estimate the APCs, AAPCs^27^, joinpoint regression modelling, parallelism test, and coincident test^28^ for ASIR^29^. Also, we used R software, version 4.3.2 (Vienna, Austria) for some data visualization like the age plots. The first year of the COVID-19 pandemic was 2020, which had a significant influence on the health system, leading to reductions in cancer screening and diagnosis, and resulted in a decrease in the 2020 incidence rates for the majority of cancer sites. As a result, the 2020 incidence data may induce bias in cancer incidence estimations, hence it was omitted from Joinpoint trends and solely displayed in graphics^30^. The ASIR of myeloma APCs were calculated by producing the best fit of least-squares regression lines on the natural logarithm of the ASIR, with the year of diagnosis as a regressor variable. The minimal number of observations between two joinpoints and from the joinpoint to either end of the data was set to two. Model selection was accomplished using the weighted Bayesian Information Criteria technique^31^. The empirical quantile method was used to obtain the 95% CIs of AAPCs^32^. The pairwise comparison using the parallelism test was used to see if the trends of the two groups were similar over time^28^. In addition, a pairwise comparison with the coincidence test was performed to see whether the rates of the two groups were identical over time.

## Results

### Myeloma

#### Overall incidence

**Figure 1:**
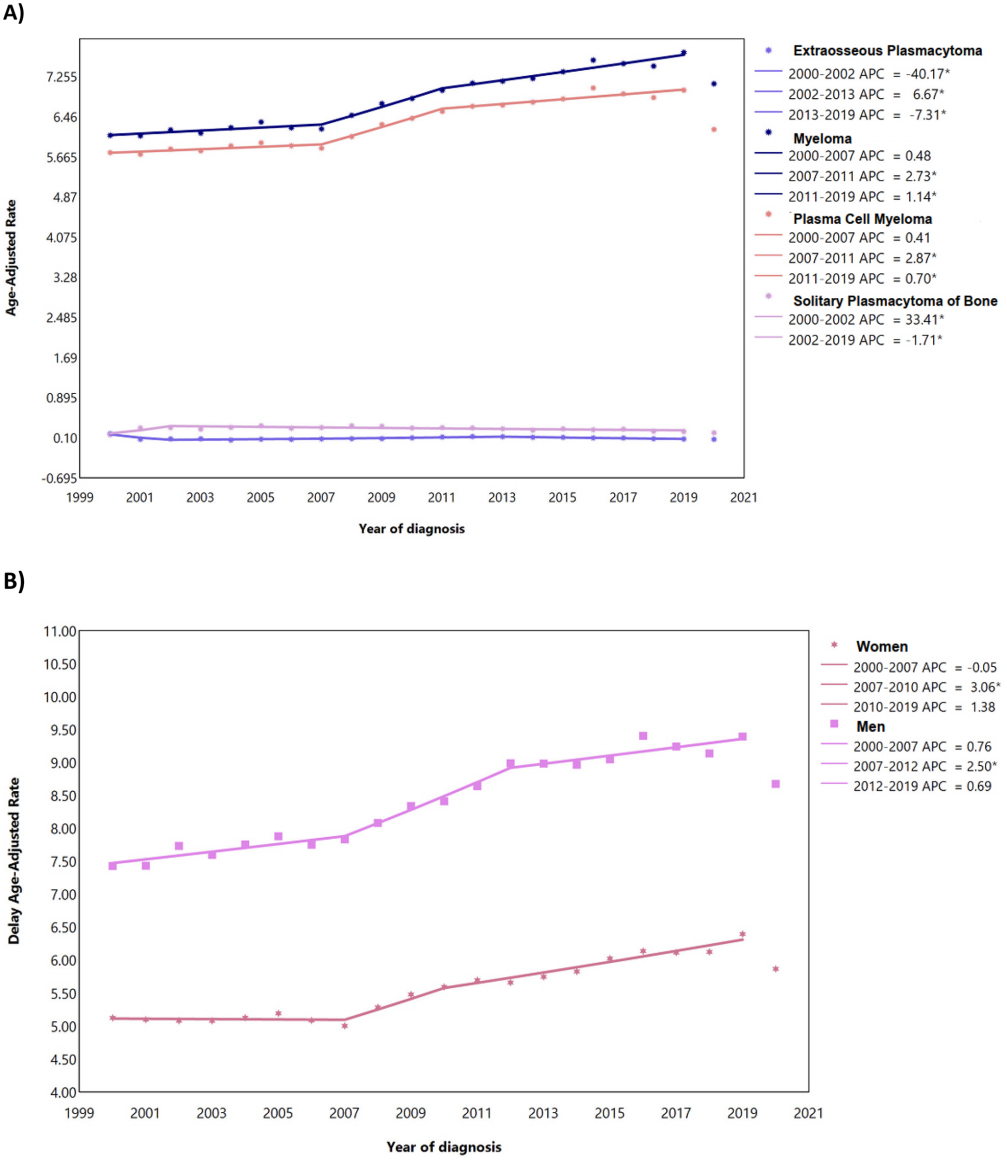

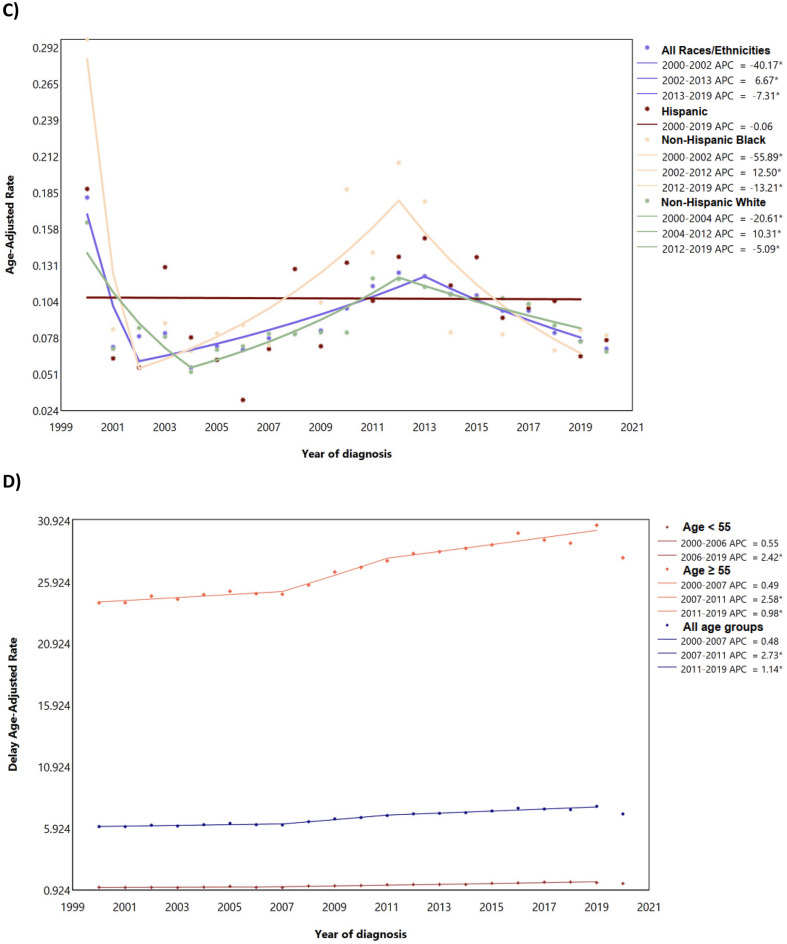
Age-adjusted incidence rate of myeloma over 2000–2019 and in 2020 in the United States, by histologic type (**A**), sex (**B**), race/ethnicity (**C**), and age (**D**). *APC* annual percent change. *Represent p-value less than 0.05.

**Figure 2:**
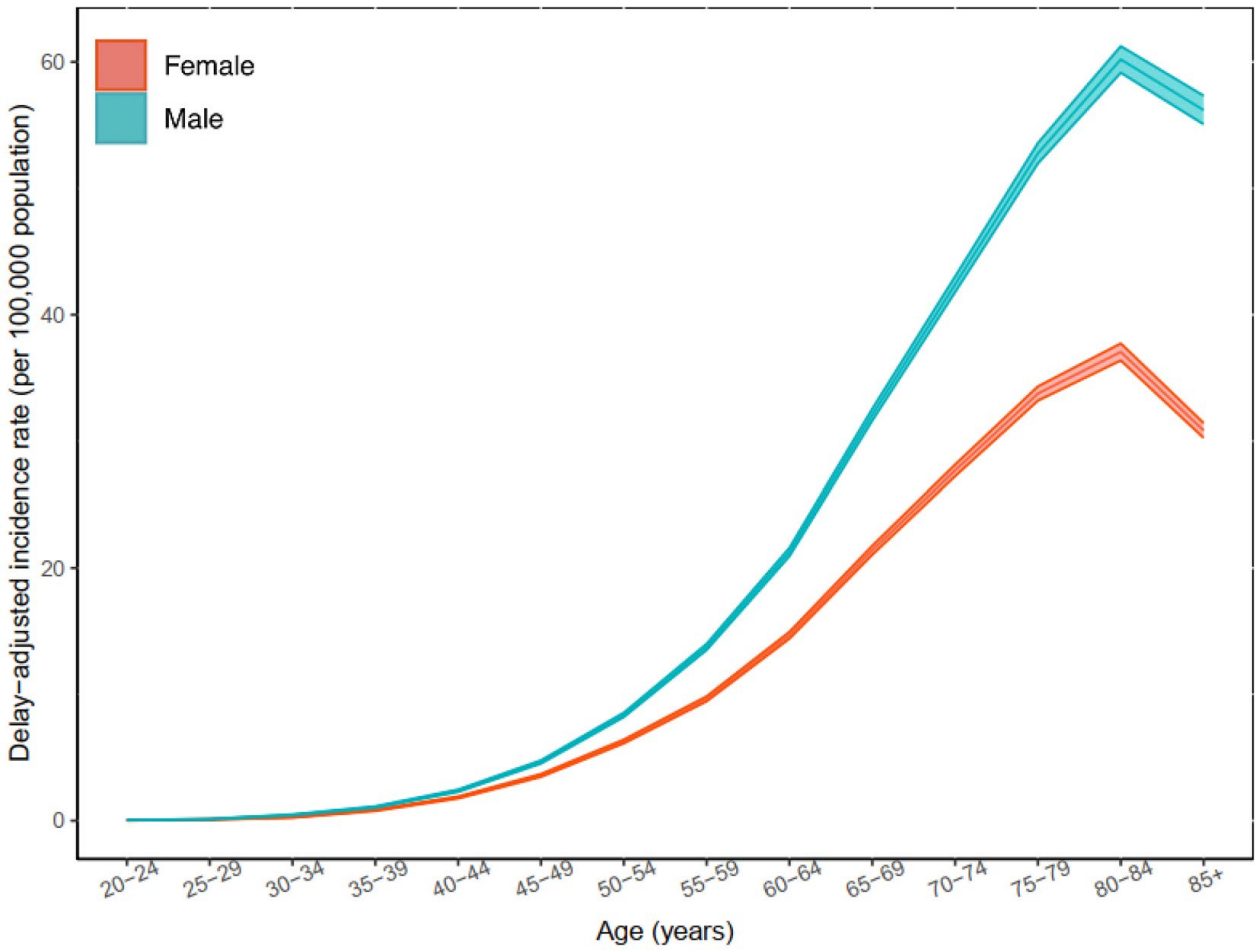
Delay-adjusted incidence rate of myeloma in the United States among males and females in each age group. Shaded areas are the confidence interval range for the point estimates. *Note*: Estimates were only provided for those with more than 16 cases.

A total of 217,049 myeloma cases were reported in the US between 2000 and 2020. The majority were males (54.85%), aged ≥ 55 years (85.64%), living in urban area (88.45%), with median income between $50,000 and $65,000 per year (28.42%) (Table 1). From 2000 to 2019, a total of 204,872 cases of myeloma were recorded in the US among all ages. The most common reported subtype was plasma cell myeloma (94.46%) (Fig. 1A). The majority of cases were observed among those aged at least 55 years (85.51%), men (54.82%), and NHWs (66.67%) (Table 2; Fig. 1B–D). From 2000 to 2019, the delayed ASIR of myeloma per 100,000 population for men and women were 8.49 (95% CI 8.43 to 8.54) and 5.58 (95% CI 5.55 to 5.62), respectively, which showed 1.19% (95% CI 1.02 to 1.33) and 1.11% (95% CI 0.91 to 1.29) increase among men and women over 2000–2019, respectively (Table 2; Fig. 1B). The delayed-adjusted incidence rate of myeloma showed a gradual rise in both sexes and peaked in 80–84 age group (Fig. 2). By race/ethnicity, NHBs had higher delayed ASIRs among men (16.62; 95% CI 16.37 to 16.87) and women (12.00; 95% CI 11.83 to 12.17), and showed the highest AAPCs for men (1.55%; 95% CI 1.26 to 1.89) and women (1.64; 95% CI 1.22 to 2.13) (Table 2; Fig. 1C). The overall trends were not parallel and identical (Table S1 and Table S2).

**Table 1:** Demographic characteristics of myeloma cases by the year of diagnosis.

Characteristics	2000–2004 N (%)	2005–2009 N (%)	2010–2014 N (%)	2015–2019 N (%)	2019–2020 N (%)	2000–2019 N (%)	2000–2020 N (%)
Sex							
Male	21,086 (53.04)	24,736 (54.54)	30,808 (55.53)	35,677 (55.49)	6752 (55.45)	112,307 (54.82)	119,059 (54.85)
Female	18,668 (46.96)	20,615 (45.46)	24,670 (44.47)	28,612 (44.51)	5425 (44.55)	92,565 (45.18)	97,990 (45.15)
Age							
< 55 year	6231 (15.67)	7003 (15.44)	7987 (14.40)	8466 (13.17)	1487 (12.21)	29,687 (14.49)	31,174 (14.36)
≥ 55 year	33,523 (84.33)	38,348 (84.56)	47,491 (85.60)	55,823 (86.83)	10,690 (87.79)	175,185 (85.51)	185,875 (85.64)
Median Income per year^a^							
< $35,000	162 (0.41)	216 (0.48)	378 (0.68)	362 (0.56)	63 (0.52)	1118 (0.55)	1181 (0.54)
$35,000–$49,999	2911 (7.32)	4109 (9.06)	6639 (11.97)	5690 (8.85)	820 (6.73)	19,349 (9.44)	20,169 (9.29)
$50,000–$64,999	9842 (24.76)	12,700 (28.00)	20,516 (36.98)	16,117 (25.07)	2508 (20.60)	59,175 (28.88)	61,683 (28.42)
≥ $65,000	26,825 (67.48)	28,316 (62.44)	27,929 (50.34)	42,116 (65.51)	8785 (72.14)	125,186 (61.10)	133,971 (61.72)
Other^b^	14 (0.04)	10 (0.02)	16 (0.03)	4 (0.01)	1 (0.01)	44 (0.02)	45 (0.02)
Area of residence							
Urban	34,506 (86.80)	39,879 (87.93)	49,251 (88.78)	57,414 (89.31)	10,926 (89.73)	181,050 (88.37)	191,976 (88.45)
Rural	5213 (13.11)	5445 (12.01)	6192 (11.16)	6843 (10.64)	1247 (10.24)	23,693 (11.56)	24,940 (11.49)
Other^c^	35 (0.09)	27 (po0.06)	35 (0.06)	32 (0.05)	4 (0.03)	129 (0.06)	133 (0.06)
Race/ethnicity							
Hispanic	4099 (10.31)	5188 (11.44)	7156 (12.90)	9124 (14.19)	1763 (2.74)	25,567 (12.48)	27,330 (12.59)
NHB	7067 (17.78)	8497 (18.74)	10,834 (19.53)	13,010 (20.24)	2413 (3.75)	39,408 (19.24)	41,821 (19.27)
NHW	27,140 (68.27)	29,722 (65.54)	34,671 (62.50)	38,471 (59.84)	7209 (11.21)	130,004 (63.46)	137,213 (63.22)
Reporting source							
Autopsy only	22 (0.06)	12 (0.03)	20 (0.04)	7 (0.01)	1 (0.01)	61 (0.03)	62 (0.03)
Death certificate only	960 (2.41)	890 (1.96)	1015 (1.83)	1187 (1.85)	248 (2.04)	4052 (1.98)	4300 (1.98)
Hospital inpatient/outpatient or clinic	35,575 (89.49)	39,460 (87.01)	47,377 (85.40)	53,711 (83.55)	10,104 (82.98)	176,123 (85.97)	186,227 (85.80)
Laboratory only (hospital or private)	538 (1.35)	559 (1.23)	1091 (1.97)	1098 (1.71)	277 (2.27)	3286 (1.60)	3563 (1.64)
Nursing/convalescent home/hospice	118 (0.30)	120 (0.26)	110 (0.20)	114 (0.18)	8 (0.07)	462 (0.23)	470 (0.22)
Other hospital outpatient unit or surgery center	1087 (2.73)	1829 (4.03)	3087 (5.56)	4392 (6.83)	886 (7.28)	10,395 (5.07)	11,281 (5.20)
Physician’s office/private medical practitioner	1296 (3.26)	1443 (3.18)	1195 (2.15)	1278 (1.99)	165 (1.36)	5212 (2.54)	5377 (2.48)
Radiation treatment or medical oncology center	158 (0.40)	1038 (2.29)	1583 (2.85)	2502 (3.89)	488 (4.01)	5281 (2.58)	5769 (2.66)

*NHW* Non-Hispanic White, *NHB* non-Hispanic Black.

^a^Median household income adjusted to 2021 inflation.

^b^Unknown, missing, and no match.

^c^Unknown, missing, no match, and Alaska or Hawaii.

**Table 2:** Counts and age-standardized rate of myeloma incidence per 100,000 and average annual percent change from 2000 to 2019 in the United States, by age, sex, and race.

	All age	Age ≥ 55	Age < 55
All races/ethnicities			
Women			
Cases (%)	92,565 (45.18)	79,645 (38.88)	12,920 (6.31)
Delayed ASIR (95% CI)	5.58 (5.55 to 5.62)	21.97 (21.81 to 22.12)	1.13 (1.11 to 1.15)
AAPC (95% CI)	1.11 (0.91 to 1.29)	0.91 (0.75 to 1.05)	2.15 (0.75 to 1.05)
Men			
Cases (%)	112,307 (54.82)	95,540 (46.63)	16,767 (8.18)
Delayed ASIR (95% CI)	8.49 (8.43 to 8.54)	34.18 (33.95 to 34.40)	1.51 (1.48 to 1.53)
AAPC (95% CI)	1.19 (1.02 to 1.33)	1.22 (1.00 to 1.54)	1.68 (1.21 to 2.18)
Hispanic			
Women			
Cases (%)	11,878 (46.46)	9454 (36.98)	2424 (9.48)
Delayed ASIR (95% CI)	5.75 (5.65 to 5.86)	22.80 (22.33 to 23.27)	1.12 (1.08 to 1.17)
AAPC (95% CI)	0.89 (0.36 to 1.86)	0.69 (0.16 to 1.49)	2.27 (1.27 to 3.49)
Men			
Cases (%)	13,689 (53.54)	10,533 (41.20)	3156 (12.34)
Delayed ASIR (95% CI)	8.19 (8.04 to 8.34)	33.04 (32.37 to 33.72)	1.44 (1.39 to 1.49)
AAPC (95% CI)	0.79 (0.42 to 1.23)	0.68 (0.27 to 1.18)	1.48 (0.58 to 2.56)
NHB			
Women			
Cases (%)	20,079 (50.95)	16,181 (41.06)	3898 (9.90)
Delayed ASIR (95% CI)	12.00 (11.83 to 12.17)	47.06 (46.33 to 47.80)	2.84 (2.75 to 2.93)
AAPC (95% CI)	1.64 (1.22 to 2.13)	1.5 (1.05 to 2.03)	2.22 (1.29 to 3.24)
Men			
Cases (%)	19,329 (49.05)	15,435 (39.16)	3894 (9.88)
Delayed ASIR (95% CI)	16.62 (16.37 to 16.87)	66.02 (64.91 to 67.14)	3.20 (3.10 to 3.30)
AAPC (95% CI)	1.55 (1.26 to 1.89)	1.99 (1.24 to 2.66)	1.55 (0.67 to 5.55)
NHW			
Women			
Cases (%)	56,072 (43.13)	50,232 (38.64)	5840 (4.50)
Delayed ASIR (95% CI)	4.78 (4.74 to 4.82)	19.25 (19.08 to 19.42)	0.85 (0.83 to 0.87)
AAPC (95% CI)	1.00 (0.78 to 1.27)	0.76 (0.54 to 1.09)	2.18 (1.60 to 2.79)
Men			
Cases (%)	73,932 (56.87)	65,149 (50.11)	8783 (6.75)
Delayed ASIR (95% CI)	7.86 (7.80 to 7.92)	32.04 (31.79 to 32.29)	1.29 (1.26 to 1.32)
AAPC (95% CI)	1.34 (1.12 to 1.58)	1.21 (0.96 To 1.58)	1.42 (0.99 to 1.85)

*NHW* Non-Hispanic White, *NHB* Non-Hispanic Black, *ASIR* Age-standardized incidence rate, *CI* Confidence interval, *AAPC* Average annual percent change.

##### Older adults (ages ≥ 55)

Over 2000–2019, a total of 175,185 cases of myeloma were diagnosed in those aged ≥ 55 years. Among older adults, plasma cell myeloma was the most common subtype (95.17%). Moreover, the majority of cases were males (54.53%) and NHWs (69.10%). Delayed ASIR per 100,000 population was 34.18 (95% CI 33.95 to 34.40) in men and 21.97 (95% CI 21.81 to 22.12) in women (Table 2). The incidence rate of myeloma in older adults increased over 2000–2019 in males and females with AAPC of 1.22 (95% CI 1.00 to 1.54) and 0.91 (95% CI 0.75 to 1.05), respectively (Table 2). Also, there was a steady increase in the incidence rate in all races, with NHB men having the highest AAPC (1.99%, 95% CI 1.24 to 2.66) (Table 2). The parallel and identical trends of myeloma among older adults are provided in Table S1 and Table S2.

##### Younger adults (ages < 55)

A total of 29,687 cases of myeloma cancer were reported over 2000–2019 among adults aged less than 55 years in the US. Most of the cases were plasma cell myeloma (90.30%). The majority of cases occurred in men (56.48%) and NHW (52.23%). The delayed ASIR per 100,000 population was 1.51 (95% CI 1.48 to 1.53) in men and 1.13 (95% CI 1.11 to 1.15) in women. Over 2000–2019, there was a higher increase in the ASIR of myeloma in women than men (AAPC: 2.15 vs. 1.68). There was an increase in incidence rates within all groups, with Hispanic women having the highest AAPC (2.27%; 95% CI 1.27 to 3.49) (Table 2). The trends of myeloma incidence over 2000–2019 among young adults in the US were not identical nor parallel (Table S1 and Table S2).

#### COVID-19 impact on myeloma

There was a significant decrease in the ASIR of myeloma across all races/ethnicities in both sexes within all age groups (AAPC: − 8.02; 95% CI − 10.43 to − 5.61) and those aged ≥ 55 (AAPC: − 8.64; 95% CI − 11.02 to − 6.25) from 2019 to November 2020. Moreover, there was a significant decrease in AAPC for females (− 8.28; 95% CI − 11.61 to − 4.96) and males (− 7.66; 95% CI − 10.78 to − 4.54) of all age groups during the COVID-19 pandemic (Table 3).

**Table 3:** Percent change in age-standardized, delay-adjusted incidence rates from 2019 to 2020, by cancer site and sex, using the November 2022 data submission.

Races/ethnicities	Sex	Age	2019 delayed ASIR (95% CI)	2020 delayed ASIR (95% CI)	AAPC (95% CI)
All	Both	All	7.73 (7.6 to 7.87)	7.11 (6.99 to 7.24)	− 8.02 (− 10.43 to − 5.61)
All	Both	< 55	1.53 (1.46 to 1.61)	1.46 (1.39 to 1.54)	− 4.58 (− 11.66 to 2.51)
All	Both	≥ 55	30.56 (30.01 to 31.12)	27.92 (27.38 to 28.46)	− 8.64 (− 11.02 to − 6.25)
All	Female	All	6.4 (6.24 to 6.57)	5.87 (5.71 to 6.03)	− 8.28 (− 11.61 to − 4.96)
All	Male	All	9.4 (9.18 to 9.62)	8.68 (8.47 to 8.89)	− 7.66 (− 10.78 to − 4.54)
All	Female	< 55	1.39 (1.29 to 1.49)	1.34 (1.24 to 1.44)	− 3.6 (− 13.39 to 6.2)
All	Female	≥ 55	24.85 (24.18 to 25.53)	22.55 (21.9 to 23.2)	− 9.26 (− 12.82 to − 5.69)
All	Male	< 55	1.68 (1.57 to 1.8)	1.59 (1.48 to 1.7)	− 5.36 (− 15 to 4.28)
All	Male	≥ 55	37.8 (36.87 to 38.74)	34.78 (33.88 to 35.7)	− 7.99 (− 11.3 to − 4.68)
Hispanic	Both	All	7.21 (6.88 to 7.56)	6.55 (6.24 to 6.88)	− 9.15 (− 15.2 to − 3.11)
Hispanic	Both	< 55	1.29 (1.16 to 1.44)	1.38 (1.24 to 1.53)	6.98 (− 8.6 to 22.55)
Hispanic	Both	≥ 55	29 (27.53 to 30.53)	25.61 (24.24 to 27.03)	− 11.69 (− 18.29 to − 5.09)
Hispanic	Female	all	6.16 (5.75 to 6.59)	5.37 (5 to 5.77)	− 12.82 (− 21.45 to − 4.2)
Hispanic	Female	< 55	1.24 (1.05 to 1.44)	1.29 (1.1 to 1.5)	4.03 (− 18.78 to 26.84)
Hispanic	Female	≥ 55	24.28 (22.5 to 26.16)	20.41 (18.8 to 22.11)	− 15.94 (− 25.2 to − 6.68)
Hispanic	Male	All	8.57 (8.02 to 9.15)	8.07 (7.54 to 8.63)	− 5.83 (− 14.78 to 3.11)
Hispanic	Male	< 55	1.35 (1.16 to 1.56)	1.47 (1.27 to 1.69)	8.89 (− 13.58 to 31.36)
Hispanic	Male	≥ 55	35.15 (32.67 to 37.76)	32.39 (30.03 to 34.88)	− 7.85 (− 17.39 to 1.69)
NHB	Both	All	16.05 (15.44 to 16.68)	14.29 (13.71 to 14.89)	− 10.97 (− 15.94 to − 5.99)
NHB	Both	< 55	3.58 (3.25 to 3.93)	3.42 (3.09 to 3.76)	− 4.47 (− 17.34 to 8.4)
NHB	Both	≥ 55	61.97 (59.39 to 64.64)	54.3 (51.86 to 56.81)	− 12.38 (− 17.81 to − 6.95)
NHB	Female	All	14.14 (13.39 to 14.93)	12.69 (11.97 to 13.43)	− 10.25 (− 17.31 to − 3.19)
NHB	Female	< 55	3.37 (2.94 to 3.84)	3.29 (2.86 to 3.77)	− 2.37 (− 21.07 to 16.32)
NHB	Female	≥ 55	53.8 (50.69 to 57.05)	47.29 (44.36 to 50.35)	− 12.1 (− 19.67 to − 4.53)
NHB	Male	All	18.78 (17.74 to 19.85)	16.73 (15.74 to 17.77)	− 10.92 (− 18.23 to − 3.6)
NHB	Male	< 55	3.82 (3.33 to 4.35)	3.56 (3.08 to 4.08)	− 6.81 (− 24.67 to 11.06)
NHB	Male	≥ 55	73.84 (69.36 to 78.53)	65.23 (60.95 to 69.71)	− 11.66 (− 19.68 to − 3.64)
NHW	Both	All	6.95 (6.8 to 7.11)	6.45 (6.3 to 6.6)	− 7.19 (− 10.27 to − 4.12)
NHW	Both	< 55	1.28 (1.18 to 1.37)	1.18 (1.09 to 1.28)	− 7.81 (− 18.23 to 2.6)
NHW	Both	≥ 55	27.85 (27.21 to 28.5)	25.83 (25.2 to 26.47)	− 7.25 (− 10.37 to − 4.14)
NHW	Female	All	5.5 (5.31 to 5.7)	5.08 (4.9 to 5.27)	− 7.64 (− 12.49 to − 2.79)
NHW	Female	< 55	1.07 (0.95 to 1.21)	0.99 (0.87 to 1.12)	− 7.48 (− 22.45 to 7.5)
NHW	Female	≥ 55	21.8 (21.03 to 22.59)	20.14 (19.39 to 20.91)	− 7.61 (− 12.32 to − 2.91)
NHW	Male	All	8.71 (8.45 to 8.97)	8.11 (7.85 to 8.36)	− 6.89 (− 10.89 to − 2.89)
NHW	Male	< 55	1.48 (1.34 to 1.63)	1.37 (1.24 to 1.52)	− 7.43 (− 20.06 to 5.2)
NHW	Male	≥ 55	35.33 (34.25 to 36.44)	32.89 (31.83 to 33.96)	− 6.91 (− 11.07 to − 2.74)

*NHW* Non-Hispanic White, *NHB* Non-Hispanic Black, *ASIR* Age-standardized incidence rate, *CI* Confidence interval, *AAPC* Average annual percent change.

### Plasma cell myeloma

#### Overall incidence

From 2000 to 2019, there were 193,530 cases of plasma cell myeloma in all age groups in the US. The majority of cases were men (54.40%), NHWs (66.52%), and aged ≥ 55 years (86.15%) (Table 3; Figs. S1, S2, S3). The ASIR per 100,000 population was 7.84 (95% CI 7.80 to 7.89) for men and 7.86 (95% CI 7.82 to 7.91) for women. The AAPCs for men and women were 1.04 (95% CI 0.90 to 1.15) and 0.89 (95% CI 0.74 to 1.02), respectively (Table 4; Fig. S1). NHB men had the highest ASIR (15.64, 95% CI 15.40 to 15.88) and NHB women had the highest AAPC (1.35; 95% CI 0.92 to 1.85) (Table 4; Fig. S2). The trends were not parallel, nor identical over 2000–2019.

**Table 4:** Counts and age-standardized rate of plasma cell myeloma incidence per 100,000 and average annual percent change from 2000 to 2019 in the United States, by age, sex, and race.

	All age	Age ≥ 55	Age < 55
All races/ethnicities			
Women			
Cases (%)	88,231 (45.60)	76,289 (39.42)	11,942 (6.17)
ASIR (95% CI)	7.86 (7.82 to 7.91)	31.97 (31.76 to 32.18 )	1.32 (1.30 to 1.34)
AAPC (95% CI)	0.89 (0.74 to 1.02)	0.68 (0.52 to 0.82)	2.25 (1.84 to 2.71)
Men			
Cases (%)	105,299 (54.40)	90,432 (46.73)	14,867 (7.68)
ASIR (95% CI)	7.84 (7.8 to 7.89)	31.87 (31.67 to 32.08)	1.32 (1.30 to 1.34)
AAPC	1.04 (0.90 to 1.15)	0.97 (0.77 to 1.14)	1.74 (1.29 to 2.23)
Hispanic			
Women			
Cases (%)	11,182 (47.00)	9017 (37.91)	2165 (9.10)
ASIR (95% CI)	5.34 (5.24 to 5.45)	21.38 (20.94 to 21.84)	0.99 (0.95 to 1.03)
AAPC (95% CI)	0.85 (0.32 to 1.50)	0.65 (0.77 to 1.25)	1.99 (0.87 to 3.38)
Men			
Cases (%)	12,607 (53.00)	9892 (41.58)	2715 (11.41)
ASIR (95% CI)	7.51 (7.37 to 7.65)	30.61 (29.97 to 31.26)	1.23 (1.18 to 1.28)
AAPC (95% CI)	0.23 (− 0. 27 to 0.75)	0.54 (0.10 to 1.09)	1.42 (055 to 2.46)
NHB			
Women			
Cases (%)	19,371 (51.18)	15,692 (41.46)	3679 (9.72)
ASIR (95% CI)	11.65 (11.48 to 11.82)	44.83 (44.12 to 45.55)	2.63 (2.55 to 2.72)
AAPC (95% CI)	1.35 (0.92 to 1.85)	1.17 (0.69 to 1.73)	2.11 (1.24 to 3.08)
Men			
Cases (%)	18,478 (48.82)	14,870 (39.29)	3608 (9.53)
ASIR (95% CI)	15.64 (15.40 to 15.88)	62.49 (61.42 to 63.56)	2.92 (2.82 to 3.01)
AAPC (95% CI)	1.27 (0.90 to 1.79)	1.21 (0.76 to 1.87)	1.52 (0.66 to 2.46)
NHW			
Women			
Cases (%)	53,343 (43.54)	47,945 (39.13)	5398 (4.41)
ASIR (95% CI)	4.49 (4.45 to 4.53)	18.14 (17.98 to 18.31)	0.78 (0.76 to 0.80)
AAPC (95% CI)	0.75 (0.53 to 1.09)	0.51 (0.26 to 0.70)	2.10 (1.48 to 2.76)
Men			
Cases (%)	69,167 (56.46)	61,463 (50.17)	7704 (6.29)
ASIR (95% CI)	7.26 (7.21 to 7.32)	29.88 (29.64 to 30.12)	1.12 (1.09 to 1.14)
AAPC (95% CI)	1.05 (0.80 to 1.34)	0.98 (0.68 to 1.31)	1. 53 (1.07 to 1.99)

*NHW* Non-Hispanic White, *NHB* Non-Hispanic Black, *ASIR* Age-standardized incidence rate, *CI* Confidence interval, *AAPC* Average annual percent change.

##### Older adults (ages ≥ 55)

A total of 166,721 cases of plasma cell myeloma were reported between 2000 and 2019 in the US among those aged ≥ 55 years. The majority of the older adults were men (54.24%) and NHWs (68.86%). The ASIR per 100,000 population was 31.87 (95% CI 31.67 to 32.08) in men and 31.97 (95% CI 31.76 to 32.18) in women. Men had higher AAPC for plasma cell myeloma incidence rate among older adults than women (0.97 vs. 0.68) (Table 4). Over 2000–2019, all races/ethnicities had significant increases in the incidence rates and NHB men had the highest increase (AAPC: 62.49; 95% CI 61.42 to 63.56) (Table 4).

##### Younger adults (ages < 55)

Over 2000–2019, a total of 26,809 cases of plasma cell myeloma were reported in the US among those aged < 55 years. The majority of them were men (55.45%) and NHWs (51.85%). The ASIR per 100,000 population was 1.32 (95% CI 1.30 to 1.34) for men and women. The AAPCs were higher in women than men (2.25 vs. 2.71) (Table 4). All groups experienced an increase between 2000 and 2019, with NHB women having the highest rate of increase (AAPC: 2.11; 95% CI 1.24 to 3.08) (Table 4).

### Extraosseous plasmacytoma

#### Overall incidence

Over 2000–2019, a total of 2864 cases of extraosseous plasmacytoma in all age groups in the US were reported. The majority of them were men (62.88%) and NHWs (71.18%), and aged ≥ 55 years (72.87%) (Table 5; Figs. S4; S5, S6). The ASIR per 100,000 population was 0.13 (95% CI 0.12 to 0.14) and 0.06 (95% CI 0.06 to 0.07) for men and women, respectively. There was a higher rate of decline in ASIR of extraosseous plasmacytoma among men than women (AAPC: − 3.41; 95% CI − 6.02 to − 0.94 for women and AAPC: − 4.10; 95% CI − 5.87 to − 2.22 for men) (Table 5; Fig. S4). NHB women had the greatest decline in ASIR of extraosseous plasmacytoma with an AAPC of − 7.67 (95% CI − 12.05 to − 3.66) (Table 5).

**Table 5:** Counts and age-standardized rate of extraosseous plasmacytoma incidence per 100,000 and average annual percent change from 2000 to 2019 in the United States, by age, sex, and race.

	All age	Age ≥ 55	Age < 55
All races/ethnicities			
Women			
Cases (%)	1063 (37.12)	798 (27.86)	265 (9.25)
ASIR (95% CI)	0.06 (0.06 to 0.07)	0.22 (0.20 to 0.23)	0.02 (0.02 to 0.03)
AAPC (95% CI)	− 3.41 (− 6.02 to − 0.94)	− 3.91 (− 6.19 to − 1.47)	0.44 (− 2.39 to 3.61)
Men			
Cases (%)	1801 (62.88)	1289 (45.01)	512 (17.88)
ASIR (95% CI)	0.13 (0.12 to 0.14)	0.44 (0.42 to 0.47)	0.05 (0.04 to 0.05)
AAPC (95% CI)	− 4.10 (− 5.87 to − 2.22)	− 3.74 (− 5.33 to − 1.84)	− 3.39 (− 7.03 to 1.41)
Hispanic			
Women			
Cases (%)	164 (37.18)	95 (21.54)	69 (15.65)
ASIR (95% CI)	0.07 (0.06 to 0.08)	0.22 (0.18 to 0.27)	0.03 (0.02 to 0.04)
AAPC (95% CI)	1.51 (− 1.23 to 5.53)	2.59 (− 1.03 to 8.47)	
Men			
Cases (%)	277 (62.82)	161 (36.51)	116 (26.30)
ASIR (95% CI)	0.14 (0.12 to 0.16)	0.48 (0.4 to 0.56)	0.05 (0.04 to 0.06)
AAPC (95% CI)	− 1.15 (− 5.61 to 4.82)		− 1.59 (− 6.13 to 3.43)
NHB			
Women			
Cases (%)	157 (46.45)	110 (32.54)	47 (13.91)
ASIR (95% CI)	0.09 (0.08 to 0.11)	0.3 (0.25 to 0.37)	0.03 (0.02 to 0.04)
AAPC (95% CI)	− 7.67 (− 12.05 to − 3.66)		
Men			
Cases (%)	181 (53.55)	118 (34.91)	63 (18.64)
ASIR (95% CI)	0.14 (0.12 to 0.17)	0.49 (0.40 to 0.59)	0.05 (0.04 to 0.07)
AAPC (95% CI)	− 4.97 (− 9.72 to − 1.58)	− 1.01 (− 14.11 to 15.24)	− 7.4 (− 11.3 to − 3.6)
NHW			
Women			
Cases (%)	677 (35.19)	548 (28.49	129 (6.70)
ASIR (95% CI)	0.06 (0.06 to 0.06)	0.21 (0.19 to 0.23)	0.02 (0.02 to 0.02)
AAPC (95% CI)	− 3.05 (− 6.15 to − 0.36)	− 3.75 (− 6.36 to − 1.19)	1.53 (− 3.94 to 7.93)
Men			
Cases (%)	1247 (64.81)	948 (49.27)	299 (15.54)
ASIR (95% CI)	0.13 (0.12 to 0.14)	0.45 (0.42 to 0.48)	0.05 (0.04 to 0.05)
AAPC (95% CI)	− 3.65 (− 5.21 to − 2.21)	− 3.72 (− 6.22 to − 1.29)	− 0.19 (− 3.52 to 3.14)

*NHW* Non-Hispanic White, *NHB* Non-Hispanic Black, *ASIR* Age-standardized incidence rate, *CI* Confidence interval, *AAPC* Average annual percent change.

##### Older adults (ages ≥ 55)

Over 2000–2019, a total of 2087 cases of extraosseous plasmacytoma among older adults in the US were reported. The majority of them were men (61.76%) and NHWs (75.55%). The ASIR per 100,000 population was 0.44 (95% CI 0.42 to 0.47) for men and 0.22 (95% CI 0.20 to 0.23) for women. Women experienced a slightly higher decline than men over 2000–2019 (AAPC: − 3.91; 95% CI − 6.19 to − 1.47 vs. AAPC: − 3.74; 95% CI − 5.33 to − 1.84). NHW women had the highest rate of decline (AAPC: − 3.75; 95% CI − 6.36 to − 1.19) (Table 5).

##### Younger adults (ages < 55)

Over 2000–2019, a total of 777 cases of extraosseous plasmacytoma among young adults in the US were reported. The majority of the patients were men (65.89%) and NHWs (59.19%). The ASIR per 100,000 population was 0.05 (95% CI 0.04 to 0.05) for men and 0.02 (95% CI 0.02 to 0.03) for women. NHB men were the only group that showed a significant decrease in AAPC (− 7.40; 95% CI − 11.30 to − 3.60) (Table 5).

### Solitary plasmacytoma of bone

#### Overall incidence

Over 2000–2019, 8478 cases of solitary plasmacytoma of bone in all age groups in the US were reported. The majority of the cases were men (61.41%), NHWs (68.52%), and aged ≥ 55 years (75.22%) (Table 6; Figs. S7, S8, and S9). The ASIR per 100,000 population was 0.38 (95% CI 0.37 to 0.39) for men and 0.20 (95% CI 0.19 to 0.20) for women. There were not significant changes in ASIR over 2000–2019 for men and women (Table 6). Only NHW women had a significant increase in AAPC (2.41; 95% CI 0.10 to 5.08) (Table 6).

**Table 6:** Counts and age-standardized rate of solitary plasmacytoma of bone incidence per 100,000 and average annual percent change from 2000 to 2019 in the United States, by age, sex, and race.

	All age	Age ≥ 55	Age < 55
All races/ethnicities			
Women			
Cases (%)	3271 (38.58)	2558 (30.17)	713 (8.41)
ASIR (95% CI)	0.20 (0.19 to 0.2)	0.69 (0.67 to 0.72)	0.06 (0.06 to 0.07)
AAPC (95% CI)	1.98 (− 0.62 to 3.72)	− 0.05 (− 1.18 to 2.34)	− 0.17 (− 2.29 to 1.96)
Men			
Cases (%)	5207 (61.42)	3819 (45.05)	1388 (16.37)
ASIR (95% CI)	0.38 (0.37 to 0.39)	1.31 (1.26 to 1.35)	0.12 (0.12 to 0.13)
AAPC (95% CI)	0.01 (− 3.22 to 3.11)	− 0.22 (− 2.15 to 1.94)	0.56 (− 0.92 to 2.01)
Hispanic			
Women			
Cases (%)	532 (39.80)	342 (25.58)	190 (14.21)
ASIR (95% CI)	0.24 (0.22 to 0.26)	0.79 (0.71 to 0.88)	0.08 (0.07 to 0.1)
AAPC (95% CI)	− 2.23 (− 4.55 to 1.16)	− 1.74 (− 4.60 to 1.70)	NA
Men			
Cases (%)	805 (60.20)	480 (35.90)	325 (24.31)
ASIR (95% CI)	0.40 (0.37 to 0.43)	1.38 (1.25 to 1.52)	0.14 (0.12 to 0.15)
AAPC (95% CI)	− 1.12 (− 4.41 to 2.9)	− 2.12 (− 4.77 to 0.80)	− 0.27 (− 3.14 to 3.12)
NHB			
Women			
Cases (%)	551 (45.13)	379 (31.04)	172 (14.08)
ASIR (95% CI)	0.32 (0.29 to 0.35)	1.04 (0.94 to 1.15)	0.12 (0.11 to 0.14)
AAPC (95% CI)	− 0.77 (− 3.76 to 2.6)	− 0.53 (− 3.76 to 3.47)	− 1.99 (− 4.34 to 0.29)
Men			
Cases (%)	670 (54.87)	447 (36.62)	223 (18.26)
ASIR (95% CI)	0.52 (0.48 to 0.56)	1.77 (1.60 to 1.95)	0.18 (0.16 to 0.21)
AAPC (95% CI)	0.40 (− 6.18 to 6.63)	0.93 (− 3.06 to 5.8)	− 2.08 (− 5.00 to 0.77)
NHW			
Women			
Cases (%)	2052 (36.84)	1739 (31.22)	313 (5.62)
ASIR (95% CI)	0.18 (0.17 to 0.19)	0.66 (0.63 to 0.69)	0.05 (0.04 to 0.05)
AAPC (95% CI)	2.41 (0.10 to 5.08)	1.73 (0.09 to 3.6)	2.15 (− 4.13 to 6.68)
Men			
Cases (%)	3518 (63.16)	2738 (49.16)	780 (14.00)
ASIR (95% CI)	0.37 (0.36 to 0.38)	1.30 (1.25 to 1.35)	0.12 (0.11 to 0.13)
AAPC (95% CI)	0.09 (− 1.29 to 1.55)	− 0.11 (− 2.11 to 2.03)	− 0.02 (− 2.89 to 1.77)

*NHW* Non-Hispanic White, *NHB* Non-Hispanic Black, *ASIR* Age-standardized incidence rate, *CI* Confidence interval, *AAPC* Average annual percent change, *NA* Not available.

##### Older adults (ages ≥ 55)

A total of 6377 cases were reported among older adults in the US over 2000–2019, with the majority of them were men (59.88%) and NHWs (73.09%). The ASIR per 100,000 population was 1.31 (95% CI 1.26 to 1.35) for men and 0.69 (95% CI 0.67 to 0.72) for women. There were not significant changes in AAPC from 2000 to 2019 for men and women. Only NHW women had a remarkable increase in AAPC (2.41; 95% CI 0.10 to 5.08) (Table 6).

##### Younger adults (ages < 55)

Over 2000–2019 a total of 2101 patients with solitary plasmacytoma of bone among young adults in the US were reported. The majority of them were men (66.06%) and NHWs (54.56%). The ASIR per 100,000 population were 0.12 (95% CI 0.12 to 0.13) for men and 0.06 (95% CI 0.06 to 0.07) for women. The AAPC did not have any significant changes by sex and race/ethnicity over the study period (Table 6).

## Discussion

This study presented the trends in incidence rates of myeloma in the US across a two-decade interval. To the best of our knowledge, this is the first US population-based study that utilized a large data integrated in the SEER program, focusing on different subtypes of myeloma to perform a comprehensive analysis of the epidemiological trends of myeloma within 2000–2020. Overall, our findings demonstrated an increasing trend of myeloma incidence in both sexes within 2000–2019. Men constituted the majority of myeloma cases (54.82%) and a male predominance was observed in all three morphological subtypes. The incidence rates of myeloma were increasing at a relatively higher rate in older aged men compared to older aged women. However, in individuals younger than 55, the incidence rates increased at a higher pace in women. While myeloma had most commonly occurred in NHWs throughout the study period, NHBs exhibited the highest increase in incidence rate both in men and women among all races/ethnicities. However, racial and ethnic disparities were evident in the trends of myeloma incidence in old and young adult participants. Accordingly, NHBs had the highest AAPC in both older men and women. However, in younger individuals, Hispanic women and NHB men exhibited the highest AAPC. Collectively, the concerning rise in the incidence rate of myeloma among younger women, specifically among young women of Hispanic ethnicity, should be carefully acknowledged when formulating public health strategies to address these worrisome patterns.

Our analysis of plasma cell myeloma incidence trends, which is the most prevalent and extensively studied subtype of myeloma, revealed a consistent pattern similar to the overall trend observed for myeloma. However, NHBs exhibited the highest rate of increase in both younger and older adults across both sexes. In a recent study by Huang et al., an increasing trend of myeloma incidence was reported globally from 2001 to 2019^33^. According to their report and consistent with our observed trends, the incidence of myeloma in the US increased both in men and women, with a higher AAPC observed for men^33^. Previous reports on the incidence rate of myeloma between 1993 and 2012 in the US, have also pointed out the increased incidence and a tendency for younger age at diagnosis; however, the only significant increases were reported to occur in NHW women and men, as well as NHB men^34^. Our findings on the other hand, suggest an increase among all races/ethnicities with higher rates of increase in NHBs. Moreover, as observed in the general trend of myeloma, the highest AAPC for myeloma in our study also belongs to the younger women, suggesting that these individuals should be considered at risk with increasing rates of myeloma.

According to our findings, extraosseous plasmacytoma was the only subtype with decreasing AAPC in both men and women. Racial disparities were also observed in the incidence rate of extraosseous plasmacytoma, as NHBs demonstrated the highest rate of decrease in both sexes across all ages. However, in older ages, NHWs witnessed the highest rate of decline in incidence rate. On the other hand, no significant changes in the incidence rate of solitary plasmacytoma of bone were observed throughout the study period, except for NHW women who exhibited significant AAPC increase, which was pronounced in the older age women. While plasmacytoma and myeloma are plasma cell disorders that share certain cytological and immunophenotypic characteristics, their tendency to affect different sites suggest that variations exist between these entities^35,36^. A previous study on the incidence of myeloma subtypes in the US has highlighted that the incidence of myeloma was 16 times higher than plasmacytoma overall, and the incidence of plasmacytoma of the bone was 40% higher than extraosseous plasmacytoma^37^. Similar to our findings, rates for plasmacytomas were higher among males, but with increasing incidence over 1992–2004^37^. A more recent study on the US population reported overall ASIRs among adults to be 0.45 for solitary plasmacytoma and 0.12 for extraosseous plasmacytoma and the incidence rates were higher in men in both diseases^38^.

In order to address the impact of the COVID-19 pandemic on the reported incidence of myeloma in the US, we analyzed the corresponding data for 2020 and the change in AAPC compared to the preceding year. Findings revealed a remarkable decrease in the ASIR of myeloma within all races and both sexes following the outbreak of the pandemic. However, the differential impact of the pandemic was observed on different age groups and races. More precisely, the decline in ASIR was highly pronounced in older age groups, as no significant changes in the ASIR of myeloma were observed in the < 55 age group across any race or sex groups. Notably, male Hispanics were the only group who were not affected by the pandemic in any of the age groups. Overall, findings suggest that the incidence of myeloma among older individuals of all races except for male Hispanics was significantly affected by the COVID-19 pandemic. Supporting these observations, a recent umbrella review has identified a substantial decrease in screening and diagnosis of several cancers during the pandemic, which was more remarkable in regions that implemented a lockdown strategy^39^. Among these cancers, myeloma is no exception as previous global reports have indicated that the number of newly diagnosed myeloma cases was reduced in 2020 compared to 2019^11^. The reduced incidence of myeloma during the COVID-19 pandemic, as observed in our study and previous reports on other cancer entities^40,41^, could be attributed to impaired screening and utilization of diagnostic measures during the pandemic^42^. The overload of medical centers with COVID-19, as well as the reluctance of patients to seek diagnostic procedures might have resulted in underreporting of cancer patients. Supporting this, studies indicate that cancer biopsies, including bone marrow biopsies which are crucial in diagnosing hematological malignancies, have remarkably decreased with the outbreak of the pandemic^43^.

The underreporting and delayed diagnosis of myeloma has resulted in increased disease burden post-pandemic. Accordingly, a report by Carmichael et al. indicated an increasing presentation of myeloma cases to emergency services with heightened rates of osseous and extra-medullary manifestations^44^. Findings of another study have also confirmed decreased survival of myeloma cases after the pandemic^11^. Moreover, reports from the early COVID-19 pandemic have also highlighted that compared with the pre-pandemic era, myeloma patients during the COVID-19 pandemic were less likely to initiate treatment or had initiated treatment later^45^. Therefore, both delayed diagnosis and postponed treatment initiation might contribute to decreased survival. The delay in treatment and changes in treatment pattern, may also cause the pandemic to leave a mark on the survival of myeloma patients in the subsequent years^44^. Although the long-term effects of these changes may not be observable for a considerable period, it is evident that the COVID-19 pandemic, and its broader influence on healthcare delivery, have led to alterations in the epidemiology and burden of individuals with myeloma and these changes could potentially have significant implications for the patients’ future prospects.

There are several strengths and limitations to this study. This is the first study to utilize the updated SEER database with robust statistical measures to provide a comprehensive US population-based study on incidence trends of myeloma. Besides analyzing the general trends of myeloma incidence, our study provided subgroup analyses on distinct pathological subtypes, including plasma cell myeloma and two less prevalent, yet critical subtypes, to better elucidate the incidence rate across various pathologic classes. Due to the unneglectable impact of the COVID-19 pandemic on cancer diagnosis, we also provided a detailed overview of changes in incidence rates of myeloma in the first year of the pandemic outbreak compared to the preceding year across all races, age groups and sexes. However, our findings might be influenced by the limitations of the SEER database. For instance, misclassification bias due to the collection of demographic data, including race and ethnicity, from various sources such as administrative databases, patient intake or provider notes is probable. Furthermore, there is uncertainty regarding whether self-identification of race and ethnicity accurately reflects ancestry or if it is primarily based on cultural factors, particularly in individuals who have mixed racial backgrounds. This ambiguity adds complexity to the interpretation of data related to race and ethnicity and should be taken into consideration when generalizing our findings. Additionally, our analysis was not sub-grouped according to race and ethnicity groups of American Indian/Alaskan Native, Asian/Pacific Islander, and Native Hawaiian due to the small sample size. Therefore, our findings may not fully represent the characteristics of individuals from these specific racial and ethnic backgrounds. The diagnostic methods of cancers can influence the incidence trends of myeloma and lead to under- or over-estimation of cancers. So, providing valid and reliable information for diagnostic methods and development of additional biomarkers data in SEER can improve the estimation of incidence of myeloma in future SEER data^46^. Also, there might be some other potential contributing factors to myeloma such as socioeconomic status that were not the aim of the study and can be considered in future studies. Moreover, data of some potential factors like occupation was not available in the current SEER iteration and can be taken into account for next SEER versions. Regarding the effects of COVID-19, there was a decreasing trend in the myeloma diagnosis by 14% in 2020 compared with 2019 at the global level^11^. The diagnosis of hematological malignancies was also decreased in the US during the COVID-19 pandemic compared with pre-pandemic period, especially due to the lockdown strategies^47^. As a result, the incidence data in 2020 was excluded from joinpoint trends. Nevertheless, it should be acknowledged as a study limitation and be considered in the interpretation of results.

## Conclusions

There was an increase in myeloma incidence in both sexes, with a highly increasing rate, particularly among younger Hispanic and NHB women between 2000 and 2019. The findings of our study also underscore the significant impact of the COVID-19 pandemic on the reported incidence rate of myeloma in 2020 across most races/ethnicities, particularly in older ages.

### Supplementary Information

Supplementary Information.

## Data Availability

The data presented in this study are available at https://seer.cancer.gov/data-software/
