# Supplementary material for: A population-based study on incidence trends of myeloma in the United States over 2000–2020
Source: Sci Rep. 2023 Nov 24;13:20705. doi: 10.1038/s41598-023-47906-y (PMC10673923; doi:10.1038/s41598-023-47906-y)
Supplement: Supplementary file 1 — Supplementary Information. [file 41598_2023_47906_MOESM1_ESM.docx]

**Table S1.** Results of the tests of parallelism for myeloma incidence rate over 2000-2019 in the United States.

| Type | Race | Age | sex | Type | Race | Age | sex | P value |
| --- | --- | --- | --- | --- | --- | --- | --- | --- |
| Cohort 1 | | | | Cohort 2 | | | |  |
| Myeloma | All | <55 | Female | Extraosseus plasmacytoma | All | <55 | Female | 0.38 |
| Myeloma | All | <55 | Female | Plasmacell myeloma | All | <55 | Female | 0.19 |
| Myeloma | All | <55 | Male | Plasmacell myeloma | All | <55 | Female | 0.28 |
| Myeloma | All | <55 | Both | Plasmacell myeloma | All | <55 | Male | 0.06 |
| Myeloma | All | <55 | Both | Plasmacell myeloma | All | <55 | Both | 0.19 |
| Myeloma | Hispanic | All | Female | Extraosseus plasmacytoma | Hispanic | All | Female | 0.49 |
| Myeloma | Hispanic | All | Male | Extraosseus plasmacytoma | Hispanic | All | Male | 0.23 |
| Myeloma | Hispanic | All | Both | Extraosseus plasmacytoma | Hispanic | All | Both | 0.08 |
| Myeloma | Hispanic | All | Both | Plasmacell myeloma | Hispanic | All | Both | 0.06 |
| Myeloma | Hispanic | ≥55 | Female | Extraosseus plasmacytoma | Hispanic | ≥55 | Female | 0.26 |
| Myeloma | Hispanic | ≥55 | Male | Plasmacell myeloma | Hispanic | ≥55 | Male | 0.25 |
| Myeloma | Hispanic | ≥55 | Both | Extraosseus plasmacytoma | Hispanic | ≥55 | Both | 0.41 |
| Myeloma | Hispanic | <55 | Male | Extraosseus plasmacytoma | Hispanic | <55 | Male | 0.13 |
| Myeloma | Hispanic | <55 | Male | Plasmacell myeloma | Hispanic | <55 | Male | 0.75 |
| Myeloma | Hispanic | <55 | Male | Solitary plasmacytoma of bone | Hispanic | <55 | Male | 0.14 |
| Myeloma | Hispanic | <55 | Both | Extraosseus plasmacytoma | Hispanic | <55 | Both | 0.11 |
| Myeloma | Hispanic | <55 | Both | Plasmacell myeloma | Hispanic | <55 | Both | 0.15 |
| Myeloma | Hispanic | <55 | Both | Solitary plasmacytoma of bone | Hispanic | <55 | Both | 0.17 |
| Myeloma | NHB | All | Female | Solitary plasmacytoma of bone | NHB | All | Female | 0.051 |
| Myeloma | NHB | ≥55 | Female | Solitary plasmacytoma of bone | NHB | ≥55 | Female | 0.09 |
| Myeloma | NHB | <55 | Male | Plasmacell myeloma | NHB | <55 | Male | 0.77 |
| Myeloma | NHW | ≥55 | Male | Plasmacell myeloma | NHW | ≥55 | Male | 0.11 |
| Myeloma | NHW | <55 | Female | Extraosseus plasmacytoma | NHW | <55 | Female | 0.61 |
| Myeloma | NHW | <55 | Male | Extraosseus plasmacytoma | NHW | <55 | Male | 0.09 |
| Myeloma | NHW | <55 | Male | Plasmacell myeloma | NHW | <55 | Male | 0.20 |
| Myeloma | NHW | <55 | Both | Extraosseus plasmacytoma | NHW | <55 | Both | 0.34 |
| Extraosseus plasmacytoma | All | All | Female | Solitary plasmacytoma of bone | All | All | Female | 0.25 |
| Extraosseus plasmacytoma | All | ≥55 | Female | Solitary plasmacytoma of bone | All | ≥55 | Female | 0.09 |
| Extraosseus plasmacytoma | All | <55 | Female | Plasmacell myeloma | All | <55 | Female | 0.20 |
| Extraosseus plasmacytoma | All | <55 | Female | Solitary plasmacytoma of bone | All | <55 | Female | 0.89 |
| Extraosseus plasmacytoma | All | <55 | Both | Plasmacell myeloma | All | <55 | Both | 0.07 |
| Extraosseus plasmacytoma | All | <55 | Both | Solitary plasmacytoma of bone | All | <55 | Both | 0.34 |
| Extraosseus plasmacytoma | Hispanic | All | Female | Plasmacell myeloma | Hispanic | All | Female | 0.46 |
| Extraosseus plasmacytoma | Hispanic | All | Female | Solitary plasmacytoma of bone | Hispanic | All | Female | 0.37 |
| Extraosseus plasmacytoma | Hispanic | All | Male | Plasmacell myeloma | Hispanic | All | Male | 0.07 |
| Extraosseus plasmacytoma | Hispanic | All | Male | Solitary plasmacytoma of bone | Hispanic | All | Male | 0.29 |
| Extraosseus plasmacytoma | Hispanic | All | Both | Plasmacell myeloma | Hispanic | All | Both | 0.08 |
| Extraosseus plasmacytoma | Hispanic | All | Both | Solitary plasmacytoma of bone | Hispanic | All | Both | 0.77 |
| Extraosseus plasmacytoma | Hispanic | ≥55 | Female | Plasmacell myeloma | Hispanic | ≥55 | Female | 0.18 |
| Extraosseus plasmacytoma | Hispanic | ≥55 | Female | Solitary plasmacytoma of bone | Hispanic | ≥55 | Female | 0.10 |
| Extraosseus plasmacytoma | Hispanic | ≥55 | Both | Plasmacell myeloma | Hispanic | ≥55 | Both | 0.12 |
| Extraosseus plasmacytoma | Hispanic | ≥55 | Both | Solitary plasmacytoma of bone | Hispanic | ≥55 | Both | 0.43 |
| Extraosseus plasmacytoma | Hispanic | <55 | Male | Plasmacell myeloma | Hispanic | <55 | Male | 0.20 |
| Extraosseus plasmacytoma | Hispanic | <55 | Male | Solitary plasmacytoma of bone | Hispanic | <55 | Male | 0.83 |
| Extraosseus plasmacytoma | Hispanic | <55 | Both | Plasmacell myeloma | Hispanic | <55 | Both | 0.15 |
| Extraosseus plasmacytoma | Hispanic | <55 | Both | Solitary plasmacytoma of bone | Hispanic | <55 | Both | 0.07 |
| Extraosseus plasmacytoma | NHB | All | Female | Solitary plasmacytoma of bone | NHB | All | Female | 0.51 |
| Extraosseus plasmacytoma | NHB | All | Male | Solitary plasmacytoma of bone | NHB | All | Male | 0.054 |
| Extraosseus plasmacytoma | NHB | All | Both | Solitary plasmacytoma of bone | NHB | All | Both | 0.07 |
| Extraosseus plasmacytoma | NHB | ≥55 | Male | Solitary plasmacytoma of bone | NHB | ≥55 | Male | 0.12 |
| Extraosseus plasmacytoma | NHB | ≥55 | Both | Solitary plasmacytoma of bone | NHB | ≥55 | Both | 0.05 |
| Extraosseus plasmacytoma | NHB | <55 | Male | Solitary plasmacytoma of bone | NHB | <55 | Male | 0.09 |
| Extraosseus plasmacytoma | NHB | <55 | Both | Solitary plasmacytoma of bone | NHB | <55 | Both | 0.30 |
| Extraosseus plasmacytoma | NHW | All | Female | Solitary plasmacytoma of bone | NHW | All | Female | 0.07 |
| Extraosseus plasmacytoma | NHW | <55 | Female | Plasmacell myeloma | NHW | <55 | Female | 0.67 |
| Extraosseus plasmacytoma | NHW | <55 | Female | Solitary plasmacytoma of bone | NHW | <55 | Female | 0.38 |
| Extraosseus plasmacytoma | NHW | <55 | Male | Plasmacell myeloma | NHW | <55 | Male | 0.10 |
| Extraosseus plasmacytoma | NHW | <55 | Both | Plasmacell myeloma | NHW | <55 | Both | 0.45 |
| Extraosseus plasmacytoma | NHW | <55 | Both | Solitary plasmacytoma of bone | NHW | <55 | Both | 0.0504 |
| Plasmacell myeloma | Hispanic | <55 | Male | Solitary plasmacytoma of bone | Hispanic | <55 | Male | 0.25 |
| Plasmacell myeloma | Hispanic | <55 | Both | Solitary plasmacytoma of bone | Hispanic | <55 | Both | 0.32 |
| Plasmacell myeloma | NHB | All | Female | Solitary plasmacytoma of bone | NHB | All | Female | 0.052 |
| Plasmacell myeloma | NHB | ≥55 | female | Solitary plasmacytoma of bone | NHB | ≥55 | female | 0.13 |

Abbreviations: NHW: Non-Hispanic White; NHB: Non-Hispanic Black.

**Table S2.** Identical trends of myeloma incidence rate over 2000-2019 in the United States.

| Type | Race | Age | sex | Type | Race | Age | sex | P value |
| --- | --- | --- | --- | --- | --- | --- | --- | --- |
| Cohort 1 | | | | Cohort 2 | | | |  |
| Myeloma | All | All | female | Myeloma | Hispanic | All | female | 0.09 |
| Myeloma | All | ≥55 | female | Myeloma | Hispanic | All | female | 0.47 |
| Solitary plasmacytoma of bone | Hispanic | ≥55 | Male | Solitary plasmacytoma of bone | white | ≥55 | Male | 0.052 |
| Extaosseus plasmacytoma | NHB | all | female | Extaosseus plasmacytoma | NHB | <55 | Both | 0.10 |
| Extaosseus plasmacytoma | NHB | <55 | male | Extaosseus plasmacytoma | NHB | <55 | Both | 0.06 |
| Extaosseus plasmacytoma | All | All | Both | Extaosseus plasmacytoma | Hispanic | All | Both | 0.86 |
| Extaosseus plasmacytoma | All | All | Both | Extaosseus plasmacytoma | NHB | All | Both | 0.10 |
| Extaosseus plasmacytoma | All | All | Both | Extaosseus plasmacytoma | NHW | All | Both | 0.81 |
| Extaosseus plasmacytoma | All | All | Male | Extaosseus plasmacytoma | Hispanic | All | male | 0.55 |
| Extaosseus plasmacytoma | All | All | Male | Extaosseus plasmacytoma | NHB | All | male | 0.14 |
| Extaosseus plasmacytoma | All | All | Male | Extaosseus plasmacytoma | NHW | All | male | 0.27 |
| Extaosseus plasmacytoma | All | All | female | Extaosseus plasmacytoma | Hispanic | All | female | 0.39 |
| Extaosseus plasmacytoma | All | All | female | Extaosseus plasmacytoma | NHW | All | female | 0.35 |
| Extaosseus plasmacytoma | All | ≥55 | Both | Extaosseus plasmacytoma | Hispanic | ≥55 | Both | 0.86 |
| Extaosseus plasmacytoma | All | ≥55 | Both | Extaosseus plasmacytoma | NHB | ≥55 | Both | 0.08 |
| Extaosseus plasmacytoma | All | ≥55 | Both | Extaosseus plasmacytoma | NHW | ≥55 | Both | 0.28 |
| Extaosseus plasmacytoma | All | ≥55 | Male | Extaosseus plasmacytoma | NHB | ≥55 | Men | 0.16 |
| Extaosseus plasmacytoma | All | ≥55 | Male | Extaosseus plasmacytoma | NHW | ≥55 | Men | 0.75 |
| Extaosseus plasmacytoma | All | ≥55 | female | Extaosseus plasmacytoma | Hispanic | ≥55 | female | 0.82 |
| Extaosseus plasmacytoma | All | ≥55 | female | Extaosseus plasmacytoma | NHW | ≥55 | female | 0.95 |
| Extaosseus plasmacytoma | All | <55 | Both | Extaosseus plasmacytoma | Hispanic | <55 | Both | 0.10 |
| Extaosseus plasmacytoma | All | <55 | Both | Extaosseus plasmacytoma | NHB | <55 | Both | 0.16 |
| Extaosseus plasmacytoma | All | <55 | Both | Extaosseus plasmacytoma | NHW | <55 | Both | 0.77 |
| Extaosseus plasmacytoma | All | <55 | Male | Extaosseus plasmacytoma | Hispanic | <55 | Male | 0.33 |
| Extaosseus plasmacytoma | All | <55 | Male | Extaosseus plasmacytoma | NHB | <55 | Male | 0.29 |
| Extaosseus plasmacytoma | All | <55 | Male | Extaosseus plasmacytoma | NHW | <55 | Male | 0.75 |
| Extaosseus plasmacytoma | All | <55 | Female | Extaosseus plasmacytoma | NHW | <55 | Female | 0.43 |
| Extaosseus plasmacytoma | Hispanic | All | Both | Extaosseus plasmacytoma | NHB | All | Both | 0.29 |
| Extaosseus plasmacytoma | Hispanic | All | Both | Extaosseus plasmacytoma | NHW | All | Both | 0.65 |
| Extaosseus plasmacytoma | Hispanic | All | Male | Extaosseus plasmacytoma | NHB | All | Male | 0.98 |
| Extaosseus plasmacytoma | Hispanic | All | Male | Extaosseus plasmacytoma | NHW | All | Male | 0.52 |
| Extaosseus plasmacytoma | Hispanic | All | Female | Extaosseus plasmacytoma | NHW | All | Female | 0.23 |
| Extaosseus plasmacytoma | Hispanic | ≥55 | Both | Extaosseus plasmacytoma | NHB | ≥55 | Both | 0.54 |
| Extaosseus plasmacytoma | Hispanic | ≥55 | Both | Extaosseus plasmacytoma | NHW | ≥55 | Both | 0.83 |
| Extaosseus plasmacytoma | Hispanic | ≥55 | Female | Extaosseus plasmacytoma | NHW | <55 | Female | 0.82 |
| Extaosseus plasmacytoma | Hispanic | <55 | Both | Extaosseus plasmacytoma | NHB | <55 | Both | 0.32 |
| Extaosseus plasmacytoma | Hispanic | <55 | Both | Extaosseus plasmacytoma | NHW | <55 | Both | 0.37 |
| Extaosseus plasmacytoma | Hispanic | <55 | Male | Extaosseus plasmacytoma | NHB | <55 | Male | 0.59 |
| Extaosseus plasmacytoma | Hispanic | <55 | Male | Extaosseus plasmacytoma | NHW | <55 | Male | 0.88 |
| Extaosseus plasmacytoma | NHB | All | Male | Extaosseus plasmacytoma | NHW | All | Male | 0.13 |
| Extaosseus plasmacytoma | NHB | ≥55 | Both | Extaosseus plasmacytoma | NHW | ≥55 | Both | 0.07 |
| Extaosseus plasmacytoma | NHB | ≥55 | Male | Extaosseus plasmacytoma | NHW | ≥55 | Male | 0.27 |
| Extaosseus plasmacytoma | NHB | <55 | Both | Extaosseus plasmacytoma | NHW | <55 | Both | 0.11 |
| Extaosseus plasmacytoma | NHB | <55 | Both | Extaosseus plasmacytoma | NHW | <55 | Both | 0.33 |


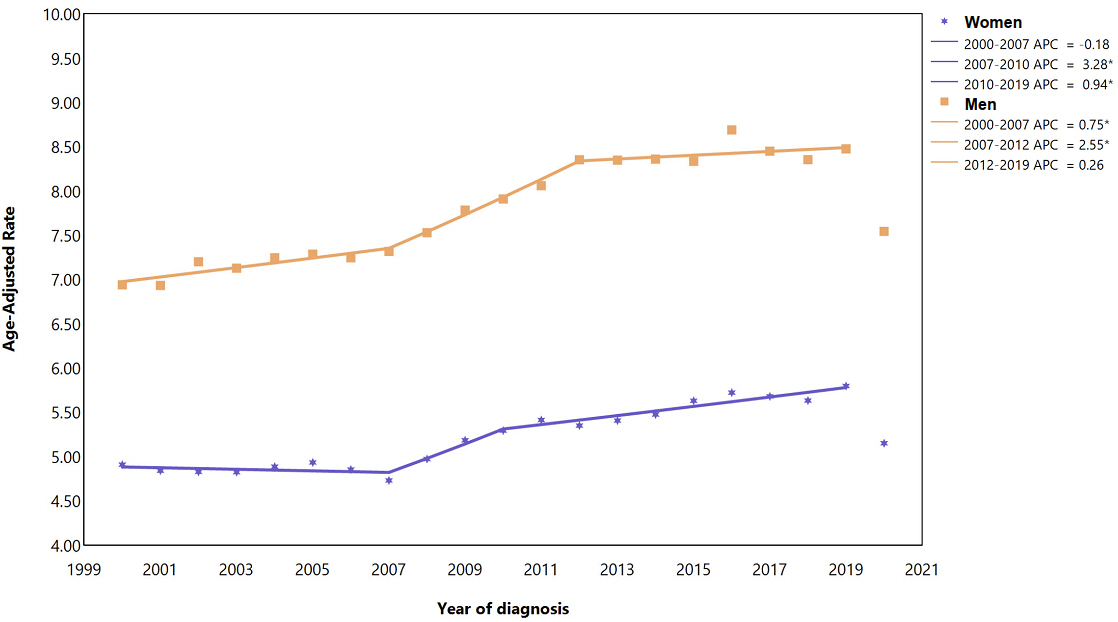


**Figure S1.** Delayed age-adjusted incidence rate of plasma cell myeloma per 100,000 people over 2000-2019 and in 2020 in the United States, by sex. APC: annual percent change. * Represent p-value less than 0.05.


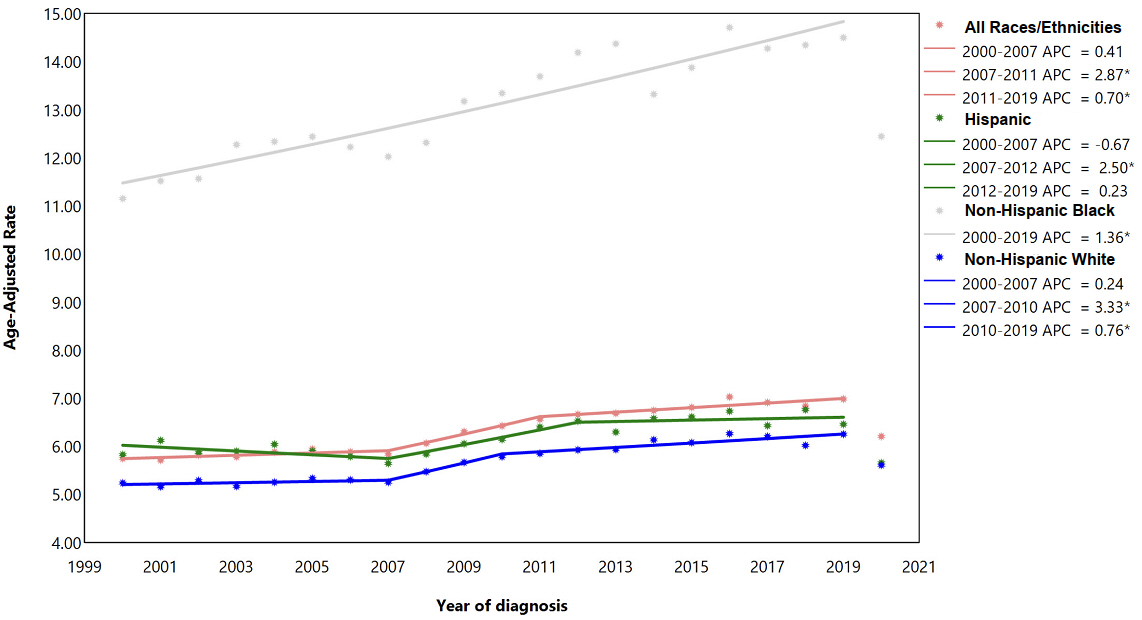


**Figure S2.** Delayed age-adjusted incidence rate of plasma cell myeloma over 2000-2019 and in 2020 in the United States, by race/ethnicity. APC: annual percent change. * Represent p-value less than 0.05.


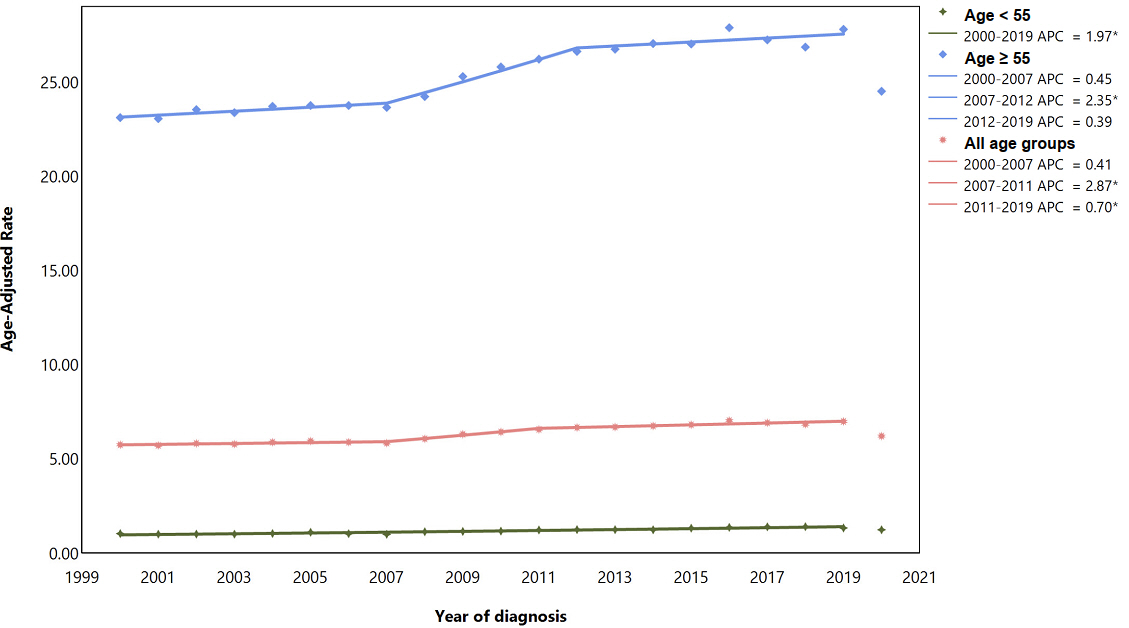


**Figure S3.** Delayed age-adjusted incidence rate of plasma cell myeloma over 2000-2019 and in 2020 in the United States, by age. APC: annual percent change. * Represent p-value less than 0.05.


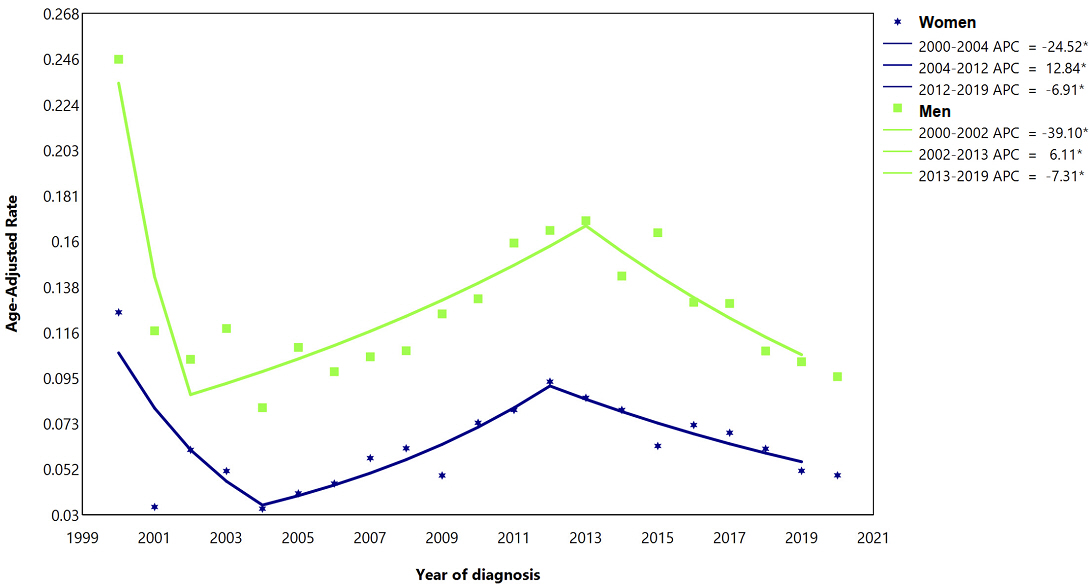


**Figure S4.** Delayed age-adjusted incidence rate of extraosseous plasmacytoma per 100,000 people over 2000-2019 and in 2020 in the United States, by sex. APC: annual percent change. * Represent p-value less than 0.05.


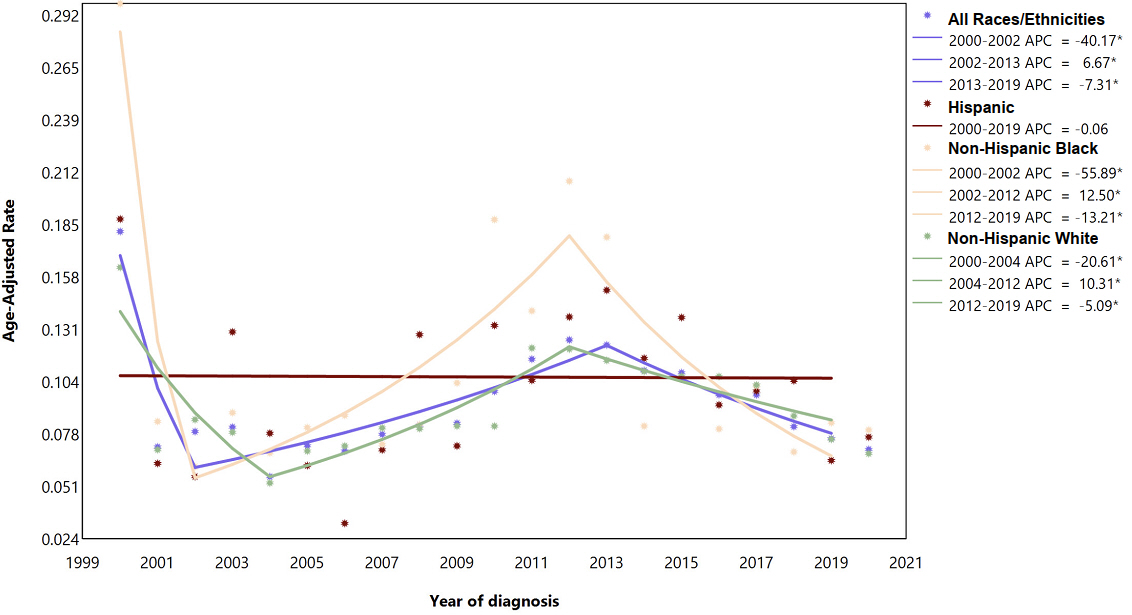


**Figure S5.** Delayed age-adjusted incidence rate of extraosseous plasmacytoma over 2000-2019 and in 2020 in the United States, by race/ethnicity. APC: annual percent change. * Represent p-value less than 0.05.


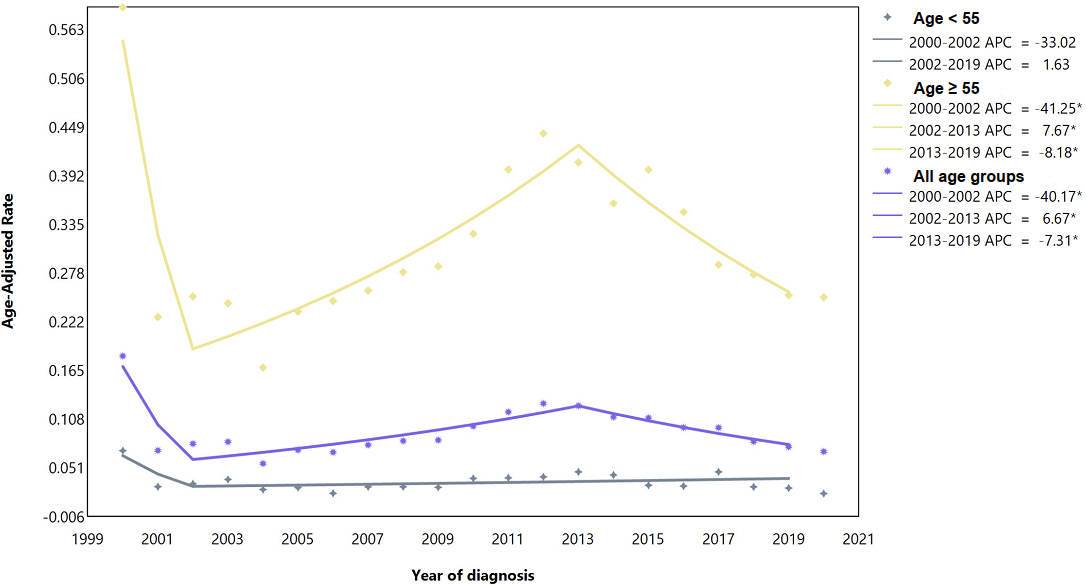


**Figure S6.** Delayed age-adjusted incidence rate of extraosseous plasmacytoma over 2000-2019 and in 2020 in the United States, by age. APC: annual percent change. * Represent p-value less than 0.05.


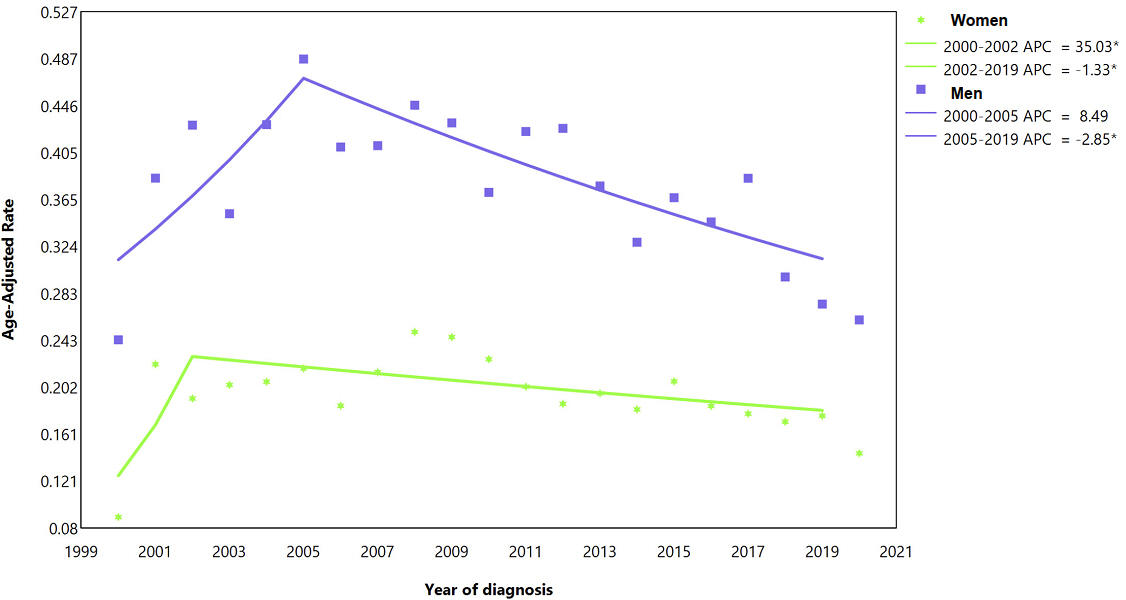


**Figure S7.** Delayed age-adjusted incidence rate of solitary plasmacytoma of bone per 100,000 people over 2000-2019 and in 2020 in the United States, by sex. APC: annual percent change. * Represent p-value less than 0.05.


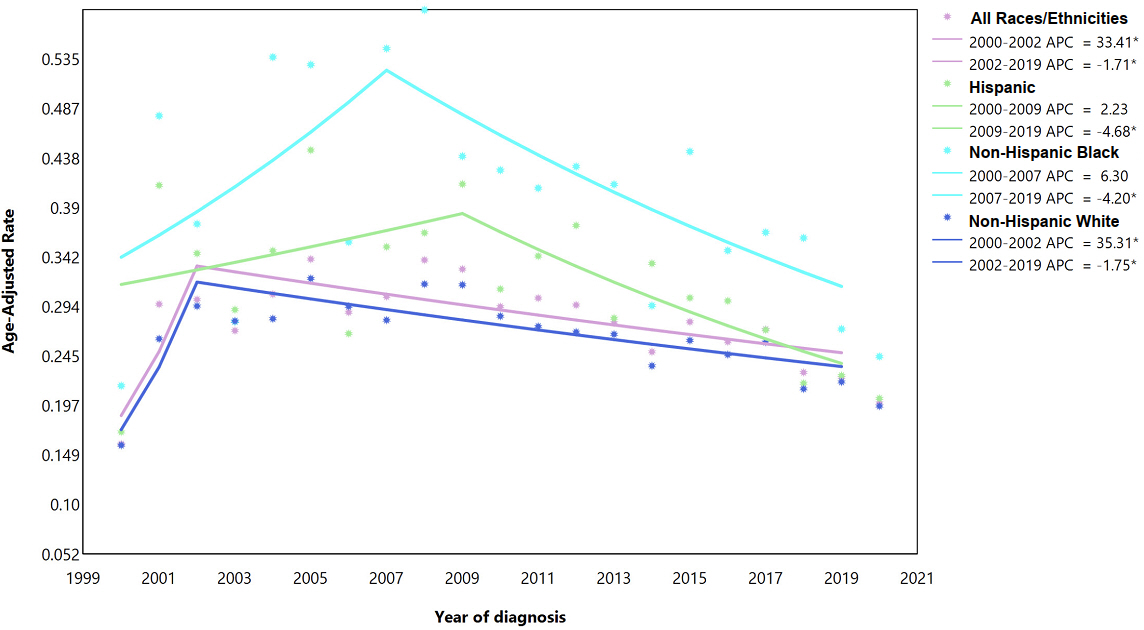


**Figure S8.** Delayed age-adjusted incidence rate of solitary plasmacytoma of bone over 2000-2019 and in 2020 in the United States, by race/ethnicity. APC: annual percent change. * Represent p-value less than 0.05.


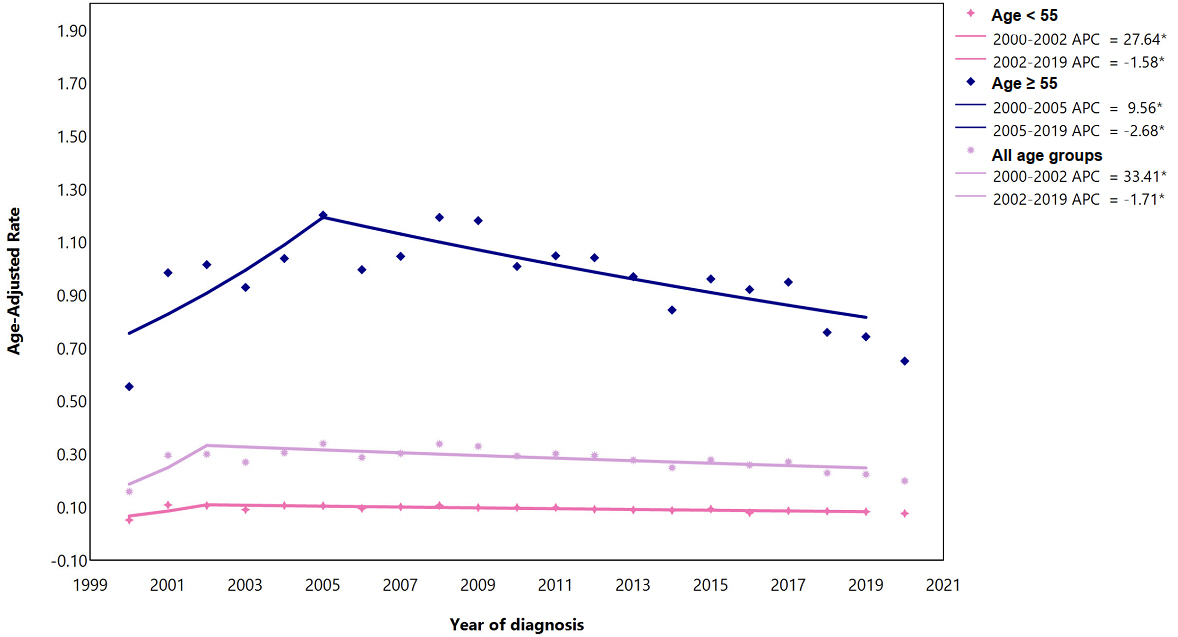


**Figure S9.** Delayed age-adjusted incidence rate of solitary plasmacytoma of bone over 2000-2019 and in 2020 in the United States, by age. APC: annual percent change. * Represent p-value less than 0.05.
